# Optimization of Pulsed Electric Field as Standalone “Green” Extraction Procedure for the Recovery of High Value-Added Compounds from Fresh Olive Leaves

**DOI:** 10.3390/antiox10101554

**Published:** 2021-09-29

**Authors:** Vasileios M. Pappas, Achillia Lakka, Dimitrios Palaiogiannis, Vassilis Athanasiadis, Eleni Bozinou, George Ntourtoglou, Dimitris P. Makris, Vassilis G. Dourtoglou, Stavros I. Lalas

**Affiliations:** 1 Department of Food Science & Nutrition, University of Thessaly, Terma N. Temponera Str., GR-43100 Karditsa, Greece; vpap@uth.gr (V.M.P.); achlakka@uth.gr (A.L.); dipaleog@med.uth.gr (D.P.); vaathanasiadis@uth.gr (V.A.); empozinou@uth.gr (E.B.); gntourtoglou@uniwa.gr (G.N.); dimitrismakris@uth.gr (D.P.M.); 2 Department of Wine, Vine & Beverage Sciences, School of Food Science, University of West Attica, Ag. Spyridonos Str., Egaleo, GR-12243 Athens, Greece; vdourt@uniwa.gr

**Keywords:** Pulsed Electric Field, fresh olive leaves, optimization, polyphenols, green, standalone

## Abstract

Olive leaves (OLL) are reported as a source of valuable antioxidants and as an agricultural by-product/waste. Thus, a twofold objective with multi-level cost and environmental benefits arises for a “green” standalone extraction technology. This study evaluates the OLL waste valorization through maximizing OLL extracts polyphenol concentration utilizing an emerging “green” non-thermal technology, Pulsed Electric Field (PEF). It also provides further insight into the PEF assistance span for static solid-liquid extraction of OLL by choosing and fine-tuning important PEF parameters such as the extraction chamber geometry, electric field strength, pulse duration, pulse period (and frequency), and extraction duration. The produced extracts were evaluated via comparison amongst them and against extracts obtained without the application of PEF. The Folin-Ciocalteu method, high-performance liquid chromatography, and differential scanning calorimetry were used to determine the extraction efficiency. The optimal PEF contribution on the total polyphenols extractability (38% increase with a 117% increase for specific metabolites) was presented for rectangular extraction chamber, 25% *v*/*v* ethanol:water solvent, pulse duration (*t*_pulse_) 2 μs, electric field strength (*E*) 0.85 kV cm^−1^, 100 μs period (*Τ*), and 15 min extraction duration (*t*_extraction_), ascertaining a significant dependence of PEF assisting extraction performance to the parameters chosen.

## 1. Introduction

Olive leaves (*Olea europaea* L.) (OLL) are listed as waste material from olive oil manufacturing and as aromatic or therapeutic herbs [1,2]. The global volume of OLL is estimated to be 12 Mt year^−1^ [3,4,5], with the majority of it produced in Europe (50%) and originating from pruning and olive oil production waste (2 Mt year^−1^).

OLL present the most abundant agricultural waste source rich in biophenols, including phenolic acids, phenolic alcohols (hydroxytyrosol and tyrosol), flavonoids (luteolin-7-*O*-glucoside, rutin, apigenin-7-*O*-glucoside, luteolin-4-*O*-glucoside), and secoiridoids (oleuropein) [2,6,7,8,9]. The composition of olive leaves varies depending on the locality, seasonality, extraction solvent, and extraction procedure used. Apart from the above constituents, oleuropein is the most prominent biophenol in olive leaf extract [8,10]. Therefore, since olive leaves are reportedly a source of high amounts in bioactive compounds and an agricultural by-product, the optimization of a “green” standalone extraction technology has multi-level benefits for the environment, the green chemical engineering technology, and the end-users (pharma, medicine, food, and nutraceutical).

OLL extracts production has been thoroughly investigated and various techniques and technologies are reported to be utilized for this purpose [11,12]. In particular, maceration, ultrasonic-assisted extraction, high pressure-assisted extraction, microwave-assisted extraction, and supercritical fluid extraction are the most popular. The limitations of the above production practices originate from their thermal processing nature (causing decomposition of thermolabile compounds while having a negative environmental impact due to high energy demand), their low extraction selectivity, and their high operating costs. As a result, greener technologies (those are more energy efficient and environmentally friendly) are required to achieve improved process efficiency [13]. Furthermore, the use of fresh leaves rather than dried leaves has lately gained popularity, owing to phytochemical thermal decomposition, particularly oxidative damage to thermolabile components, during plant drying [14,15].

Pulsed Electric Field (PEF) is a relatively recent, yet emerging eco-extraction technology of biologically active compounds (BACs). PEF has minimum environmental impact since it has minimum energy requirements and a non-thermal approach. It may be used in batch and continuous flow applications and meets the standards of green chemical engineering for long-term production systems [16]. The degree to which PEF is effective in assisting the extraction of intracellular solutes from fresh plant materials is determined from the degree to which electroporation is achieved (electrically induced formation of aqueous pores in the lipid bilayer) in a periodical and non-destructive manner for the cell under the influence of the induced transmembrane voltage by the PEF application. Electroporation occurs in such a way that components of interest migrate from the inner portion of the cell envelope to the outer part, where the solvent transports them away in solution, resulting in an increase in mass transfer and hence yield improvement for the solid-liquid extraction. The electric field strength (*E*), pulse shape, pulse duration (*t*_pulse_), pulse period (*T*) or frequency (*f*), and total extraction duration (*t*_extraction_) are among the parameters that must be fine-tuned when using PEF technology to improve extraction of a specific solid-liquid system [17].

PEF’s influence on microorganism inactivation at high specific energy input levels [18,19], pretreatment of a variety of plant materials for downstream processes at low to moderate specific energy input levels [10,20,21,22,23,24,25,26,27], and even direct extraction of plant material are well documented [28,29,30]. The first reported attempt to use PEF technology as a primary extraction enhancement of high value-adding compounds from plant cell suspension cultures was by Brodelius et al. [31]. Other researchers have introduced electric field treatment for the aging acceleration of young wine, through flavor compounds extraction from wood [32,33]. Recently, Ntourtoglou et al. [34] revealed that PEF assisted extraction resulted in an increase of bitter hops acids extraction rate by 20%. Finally, Tsapou et al. [35] applied pulsed electric field (PEF) to beer wort enriched with flax seeds to fine-tune the production of phenolic aromas in beer, also by electroporation and achieved production efficiency up to 120%.

PEF technology is now being fine-tuned as the principal standalone extraction method for BACs extraction from plant material based on biomass characteristics, composition, and degree of comminution. However, there is still limited knowledge and understanding for the complex multi-parameter phenomena involved in the root cause analysis of the mechanisms that occur and affect the extraction rates of components of interest. As a result, there is lots of room for technological advancement, invention, and discovery. Usually, PEF is used as a preparative step before extraction that utilizes other techniques (such as ultrasound). In this work, the method proposed is a standalone extraction method for valuable bio-functional components that can be used in a simple, “green” and long-term manner. Furthermore, using PEF with green aqueous organic solvent mixes instead of pure water is a relatively novel method that can improve extraction yield even further. Given that each plant material exhibits a different behavior when trying to isolate one or more of its active ingredients, it is always necessary to prove with a study that the new method is applicable. It is worth mentioning that the results or the conditions used in one study concerning a specific plant material do not necessarily apply to another.

In our previous study [36], we presented the initial results of our ongoing work on PEF and proposed this technique as a standalone extraction method of valuable bio-functional components, which can be applied in a simple “green” sustainable way for the extraction of OLL. We used an electric field of 1 kV cm^−1^ with a pulse duration of 10 or 100 μs under a period of 1000 μs for 30 min. Extraction solvents included water, ethanol, and combinations of the two.

The present study aimed to provide further insight on the PEF for a standalone solid–liquid static extraction of OLL under the target of maximizing extracts’ polyphenol concentration. Towards that end, we performed a process optimization study by first choosing the best extraction chamber geometry and then by fine-tuning important PEF parameters such as the electric field strength (*E*), pulse shape, pulse duration (*t*_pulse_), pulse period (*T*), and the total extraction duration (*t*_extraction_). The average particle size, solvent type, pH, solvent to OLL ratio, and extraction temperature were kept constant throughout this study based on screening and findings from previous studies of our group [36,37]. The produced extracts were evaluated via comparison amongst them and against extracts obtained without the application of PEF. The Folin–Ciocalteu method (for total polyphenol content), high-performance liquid chromatography (HPLC), and differential scanning calorimetry (DSC) were used to assess the extraction effectiveness.

The novelty of this work lies upon the optimization of PEF, by choosing and fine-tuning important PEF parameters such as the extraction chamber geometry, electric field strength, pulse duration, pulse period (and frequency), and extraction duration, for the static solid-liquid extraction of olive leaves BACs (including the thermolabile compounds) in green solvents (pure water, pure ethanol; and their mixtures), using fresh plant material instead of dried. To the best of our knowledge there are no reports with such a holistic approach for PEF as a standalone OLL extraction. The potential of the PEF technology application in the proposed way paves the road for industrial applications (after appropriate scale-up and further fine-tuning) and new scientific discoveries.

## 2. Materials and Methods

### 2.1. Chemicals

HPLC grade solvents were utilized for liquid chromatography. Acetonitrile and formic acid (99%) were purchased from Carlo Erba (Val de Reuil, France). Sodium carbonate anhydrous (99%) and gallic acid monohydrate were purchased from Penta (Prague, Czech Republic). Luteolin-7-*O*-glucoside, apigenin, rutin hydrate and oleuropein were purchased from Sigma-Aldrich (St. Louis, Burlington, MA, USA). Ethanol (99.8%) and Folin–Ciocalteu reagent were purchased from Panreac (Barcelona, Spain).

### 2.2. Plant Material, Handling and Sample Preparation

The OLL utilized in this study was again harvested from a 30-year-old olive tree (*Olea europaea* L., cv. Chondrolia Chalkidikis), in the Karditsa Region of Greece (at 39°21′46″ N and 21°55′05″ E, with an elevation of 108 m, according to Google Earth version 9.124.0.1, Google, Inc., Mountain View, CA, USA). After the harvesting season, the experiments were done from February 2 to February 11, 2021. The average temperature was between 9 °C and 15 °C, with an average relative humidity of 80%. Early in the morning of each experimental series day, the OLL were collected as branches and delivered to the lab 10 min later for rapid processing. After the branch removal, the leaves were completely cleaned with tap water and dried with filter paper at ambient temperature (22 °C) until no extra moisture was present on the leaves’ surface. To achieve homogeneity of the pulverization outcome and minimal temperature rise, the leaves were crushed for 2 min in a blender Camry CR 4071 (Adler Europe Group ul., Warszawa, Poland) under identical shear input and batch quantities before each extraction attempt. The latter resulted in powders with an approximate average particle diameter of about 0.8 mm (*d*_10_ = 370 μm, *d*_50_ = 760 μm, *d*_90_ = 1130 μm) as determined by sieve analysis.

**Figure 1:**
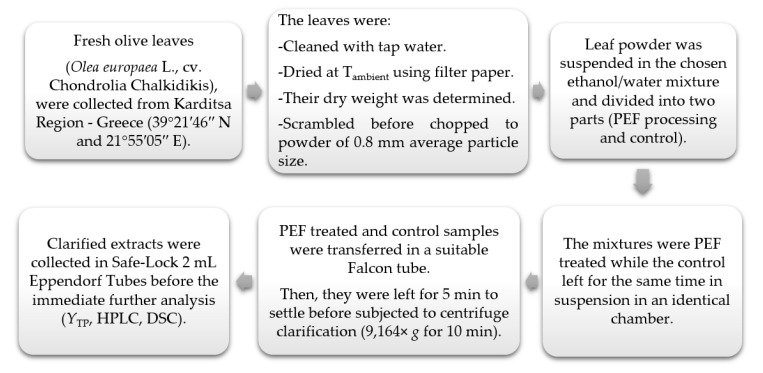
Plant material processing steps. Abbreviations: PEF (Pulsed Electric Field); *Y*_TP_ (Total Polyphenol Content); HPLC (High Performance Liquid Chromatography); DSC (Differential Scanning Calorimetry).

The solvent was added to the freshly cut finely powdered OLL after grinding, and the mixture was then placed into the PEF treatment chamber. The raw material to solvent ratio was 1:3 (*w*/*v*) in all extraction runs, with 18 g of freshly cut and finely powdered OLL and 54 mL of solvent. All experiments occurred at ambient temperature (22 °C). The suspensions were separated from the plant material, which was subsequently discarded, after each extraction. The extracts were transferred to a suitable Falcon tube and allowed for 5 min before centrifuge clarified (9164× *g* for 10 min at ambient temperature). The clarified extracts were collected in Safe-Lock 2 mL Eppendorf tubes before the immediate further analysis (*Y*_TP_, HPLC, and DSC). All produced PEF treated extracts were evaluated via comparison amongst them and against control extracts (obtained without the application of PEF). Triplicates of each extraction run were performed. An infrared thermometer (GM300, Benetech, Shenzhen Jumaoyuan Science and Technology Co., Ltd., Shenzhen, China) was used to measure the temperature of the treatment chamber contents before and after each extraction run. The temperature increments owing to the treatment in all PEF assisted extraction runs never exceeded a ΔT of 1 °C. The synopsis of the plant material processing steps is presented in Figure 1. 

### 2.3. Dry Matter/Water Content Determination

For the determination of the water content of each batch of pulverized leaves, an adequate quantity was weighed before and after drying until constant weight, at 85 °C using an oven (Binder BD56, Bohemia, NY, USA). The following Equation (1) was then used for the calculation of the percentage of moisture and volatiles content [37]:

(1) $$\%\;\mathrm{Moisture}\;\mathrm{and}\;\mathrm{volatiles}\;\mathrm{content}=\frac{W_{\mathrm{BD}}-W_{\mathrm{AD}}}{W_{\mathrm{BD}}}\times \;100\;$$

where *W_BD_* is the weight (g) of pulverized leaves before drying, and *W_AD_* is the weight (g) of pulverized leaves after drying. The leaves had a moisture and volatiles content of about 50% (*w*/*w*). Equation (2) was used to get the dry matter (g) determination for each sample [37]:

(2) $${\mathrm{Dry}\;\mathrm{matter}=W}_{S\;}-\left(W_{S}\;\times \;\%\;\mathrm{Moisture}\;\mathrm{and}\;\mathrm{volatiles}\;\mathrm{content}\right),$$

where *W_S_* is the weight (g) of pulverized leaves without drying used as sample.

### 2.4. PEF System and Calculus

The PEF system used is the same with the one presented earlier by Pappas et al. [36]. It is a static bench-scale system that includes a high voltage (0.1–25 kV) power generator, a 25 MHz Function/Arbitrary Waveform Generator, a tailored electronic switch circuit (series of Insulated gate bipolar transistors—IGBTs), and two twin sets of custom-made stainless-steel treatment chambers, one rectangular and one cylindrical of similar volumes. In particular, the rectangular consists of two identical flat parallel stainless-steel plates 10 cm × 10 cm separated by a “Π” shaped Teflon single piece that functions as an insulator at a regular spacing of 1 cm [36]. The cylindrical stainless-steel treatment chamber (internal diameter 3 cm and length 17 cm), including a solid stainless-steel concentric electrode (diameter 1 cm and length 17 cm), was fastened to Teflon screw caps in both ends, where the positive electrode was attached at the concentric electrode while the negative return at the outer layer of the treatment chamber. Both chambers had effective volumes equal to 80 mL.

The set of equations used for the calculation of the electric field strength (*E*), the total PEF treatment time (*t*_PEFtreatment_), and the specific energy input *W*_spec_ (kJ kg^−1^), are adequately described in our previous work [36]. The pulse generator provided unipolar, rectangular-shaped pulses, with pulse duration (*t*_pulse_) varying between 1, 2, 5, 10, and 20 μs under a period (*T*) of 100, 500, and 1000 μs, for a specific number of pulses (*N*) defined by the extraction duration (*t*_extraction_) and the period (*T*).

For the intrinsic property of conductivity, a 743 Rancimat (Metrohm UK Ltd., Cheshire WA7 1LZ, UK) was utilized, giving a measurement of 0.8 μs cm^−1^ for our solvent of choice (25% *v*/*v* EtOH:H_2_O), and an average of 691 μs cm^−1^ for the extracts.

### 2.5. Experimental Design

The key PEF parameters were screened in depth in order to determine the best PEF parameters for the given system (plant material and solvent) in order to maximize the extracts’ polyphenolic content. The permeability regulating parameters, which include field intensity (*E*), pulse duration (*t*_pulse_), and the pulse period (*T*) for a specific extraction duration (*t*_extraction_), were chosen as the major PEF parameters. Extracts of OLL treated with PEF showed greater concentrations of polyphenols at PEF pulse duration (*t*_pulse_) of 10 μs, pulse period (*T*) of 1000 μs, electric field strength (*E*) of 1 kV cm^−1^, and extraction time (*t*_extraction_) of 30 min in aqueous ethanol, 25% *v*/*v*, as reported by Pappas et al. [36].

The study design (process optimization sections and parameters) was progress based and structured in a way that the result and conclusion from each section was adopted as input to the following section. The resulting list of experiments are presented in Table 1, and included the following sections:

#### 2.5.1. Experimental Section 1. Determination of the Optimal Extraction Chamber (Cell) Geometry

As a starting point of this optimization study, the potential of whether the chamber geometry is a key parameter in the static extraction behavior was evaluated. For this reason, two different chamber geometries were tested; A cylindrical and a rectangular one (described in Section 2.4).

#### 2.5.2. Experimental Section 2. Determination of the Optimal Electric Field Strength 

Based on the outcome of Exp. Section 1, the optimal electric field strength was defined. Three levels of moderate intensity were utilized, namely 1, 0.85, and 0.7 kV cm^−1^.

#### 2.5.3. Experimental Section 3a. Determination of the Optimal PEF Pulse Duration

Based on the outcomes of Exp. Sections 1 and 2, the definition of the optimal pulse duration using a rectangular unipolar step-change while keeping a specific (*t*_pulse_:*T*) analogy equal to 1:100 took place. At this section, three values were used for the *t*_pulse_, namely 10, 5, and 1 μs.

#### 2.5.4. Experimental Section 3b. Determination of the Optimal PEF Pulse Period

Based on the outcomes of the Exp. Sections 1 and 2, the optimal pulse duration using a rectangular unipolar step-change while altering pulse time to period analogy was defined. Here, the three sets of (*t*_pulse_:*T*) values used were (1:1000), (5:1000), and (20:1000).

#### 2.5.5. Experimental Section 4. Determination of the Optimal Extraction Time

Based on the outcomes of Sections 1, 2 and 3, followed the definition of the optimal extraction duration amongst three values, namely 30, 15, and 10 min.

#### 2.5.6. Experimental Section 5. Verification 

Confirmation and re-evaluation of the best cases for the optimal extraction duration.

### 2.6. Total Polyphenol Content of Extracts

The method was adopted by Lakka et al. [38] who employed a validated protocol (using Folin-Ciocalteu reagent) to analyze the results, which were reported as mg of gallic acid equivalents (GAE) per gram of dry weight (dw) based on the reference gallic acid calibration curve (10–80 mg L^−1^) generated for this study. The total polyphenol yield (*Y*_TP_) was calculated using the Equation (3):

(3) $$Y_{\mathrm{TP}}\;(\mathrm{mg}\;\mathrm{GAE}\;g^{-1}\;\mathrm{of}\;\mathrm{dw})=\frac{C_{\mathrm{TP}}\;\times \;V}{w}$$

where *C*_TP_ is the extract’s total polyphenol concentration (mg L^−1^), *V* is the volume of the extraction medium (L), and *w* is the plant material’s dry weight (g).

### 2.7. High-Performance Liquid Chromatography (HPLC)

The extracts prepared during Exp. Section 5 (21, 22, 23 and 24) were analyzed using a method adopted by Kaltsa et al. [7]. A Shimadzu CBM-20A liquid chromatograph (Shimadzu Europa GmbH, Duisburg, Germany), coupled to a Shimadzu SPD-M20A photodiode-array detector (PDA), and interfaced by Shimadzu LC solution software, was used for chromatographic studies. A Phenomenex Luna C18(2) (100 Å, 5 μm, 4.6 × 250 mm) column was employed (Phenomenex, Inc., Torrance, CA, USA). The temperature of the analysis was adjusted to 40 °C and the eluents used were (A) 0.5% aqueous formic acid and (B) 0.5% formic acid in acetonitrile/water (6:4). The injection volume was 20 μL and the flow rate was 1 mL min^−1^. The gradient elution program was as follows: 100% A to 60% A in 40 min; 60% A to 50% A in 10 min; 50% A to 30% A in 10 min, which was kept constant for another 10 min. The equations of the standards calibration curves were used to accomplish quantification.

### 2.8. Differential Scanning Calorimetry (DSC)

After evaporating the solvents with a rotary evaporator (Laborota 4000, Heidolph, Schwabach, Germany), antioxidant activity was estimated using the DSC technique as described by Pappas et al. [36]. A Perkin Elmer Diamond DSC was used to make the measurements (PerkinElmer Inc., Shelton, CT, USA). As a purge gas, oxygen was used. In short, empty hermetically sealed pans were used as control, while 4–5 mg of each sample was placed in DSC aluminum pans with hole (1 mm in diameter) in the lids to allow the oxygen stream to reach the sample. Hold for 1 min at 40 °C, heat from 40 to 200 °C (40 °C min^−1^), and finally heat from 200 to 580 °C (20 °C min^−1^) were the temperature program used. The starting temperature of oxidation is determined by the onset temperature of the oxidation peak (*T*_max_).

### 2.9. Statistical Analysis

All extraction series and spectrophotometric measurements were done in triplicate, with the average and standard deviation (SD) of three separate experiments shown. The results were statistically analyzed using Microsoft Excel 2019 (Redmond, WA, USA) software. The statistical significance (at *p* < 0.05) between mean values was determined using one-way analysis of variance (ANOVA).

## 3. Results

This study focused on the PEF process optimization towards maximizing BACs content of OLL in green solvents, based on our previous work [36]. To find the optimal conditions, the variables tested were as follows; two different chamber geometries, three different electric field strengths, various pulse durations and pulse periods, and three different extraction times. A final verification section assisted in concluding the optimal conditions. The effect of the above-chosen parameters input values differentiation on the total polyphenolic composition between the control and PEF treated samples transpired via the Folin–Ciocalteu method towards extraction efficiency optimization. Further analysis of the polyphenolic profile was carried out with HPLC-PDA for the control and the samples produced under optimal conditions to determine the extraction efficiency enhancement, thus ascertain any selectivity of the main components extracted. In addition, an estimation of the oxidation resistance of the extracts was done by utilizing the DSC technique. 

### 3.1. Experimental Section 1 (Exp. Series 1–4)—Rectangular vs. Cylindrical Extraction Chamber 

The design of the treatment chambers is critical to the development of PEF technology since they hold the sample material during PEF application and house the discharging electrodes. A treatment chamber is made up of two electrodes that are held in place by an insulating substance that also serves as a container for sample materials. The electrode configurations that can be used are parallel plates, parallel wires, concentric cylinders, and a rod plate [39]. Parallel plates are the most practical choice because they create a homogeneous electric field strength distribution over a large useful area. Concentric cylinders, on the other hand, provide a smooth and uniform product flow and are popular in industrial applications.

For the first section, the treatment parameters for extraction of the finely ground OLL was an electric field of 1 kV cm^−1^, a pulse duration of 10 μs, and a pulse period of 1000 μs for a 30 min extraction duration. For the treatment chamber, two different geometries, rectangular and cylindrical were chosen. The highest percentage increase in *Y*_TP_ between PEF and control samples transpired with the rectangular chamber. The results (Table 2, Exp. Series 1–4) showed that the PEF treatment into the rectangular chamber led to a 33.8% increase, while into the cylindrical utilization resulted in a 16.0%, both significant (*p* < 0.05) compared to the control samples. In particular, when PEF was applied, the *Y*_TP_ by the rectangular chamber appeared to be 24.98 ± 0.56 mg GAE g^−1^ dw, while the one from the cylindrical chamber reached 16.66 ± 1.55 mg GAE g^−1^ dw. Except for the lower *Y*_TP_ measured for the case of the cylindrical chamber versus the rectangular one, a lower percentage increase was reached when comparing the PEF treated sample in cylindrical chamber to the control. It appears that the uniformity of the field in the rectangular geometry is dominant. Thus, the rectangular chamber was chosen as the optimal geometry to continue the optimization study.

### 3.2. Experimental Section 2 (Exp. Series 5–8)—Optimal Electric Field Strength 

All the cells in the sample are exposed to the same electric field in uniform electric field chambers, which is beneficial for electroporation. If the field strength is enough and close to the optimal value, high intracellular compound extraction yields are feasible. However, given that optimum extraction yields can fall significantly above or below the optimum field strength, the optimum value for the electric field strength must always be determined through structured experimental design.

Based on the literature [40], it was decided to screen the electric field for an optimal effect on the extraction of bioactive compounds from OLL at the range of 0.7 to 1 kV cm^−1^, keeping the level of specific energy input bellow 5 kJ kg^−1^. Thus, for this optimization section (Exp. Section 2), three different input values for the electric field strength were tested, namely 1, 0.85, and 0.7 kV cm−1. The field strength of 1 kV cm^−1^ resulted in an increase of 29.1% in *Y*_TP_. In particular, the results (Table 2, Exp. Series 5–8) showed that the specific PEF sample gave a *Y*_TP_ of 24.80 ± 1.36 mg GAE g^−1^ dw while the control sample 19.20 ± 0.66 mg GAE g^−1^ dw. The application of field strengths of 0.85 and 0.7 kV cm^−1^ resulted in higher percentage significant (*p* < 0.05) increases (38.1% and 36.9%, respectively). The highest increase was observed for the case of 0.85 kV cm^−1^ field strength and is in line with previous studies. Although, it was not significantly different than that of 0.7 kV cm^−1^, it was selected to continue the optimization study.

### 3.3. Experimental Section 3a and 3b—Optimal PEF Pulse Duration and Period (Exp. Series 9–12 and 13–16)

For Exp. Section 3 of the optimization study, different pulse durations or periods were examined, thus altering the cell membrane relaxation time or the specific energy applied to the sample towards revealing the best combination for the OLL extraction. For this section, based on the outcome of Exp. Sections 1 and 2, the starting point was the rectangular chamber and electric field strength of 0.85 kV cm^−1^ for an extraction duration of 30 min. The targeted research inquiry of this optimization section was twofold. For the first part, the changes of *t*_pulse_ and *T* followed a constant ratio of 1:100, while for the second part, we experimented with different *t*_pulse_ under a fixed *T*.

For Exp. Section 3a (Table 2, Exp. Series 9–12), the highest percentage increase between PEF and the control sample was achieved when a pulse duration of 1 μs and a pulse period of 100 μs (31.2%) was applied. In particular, the *Y*_TP_ for the extract produced with *t*_pulse_ 1 μs and *T* 100 μs was 24.91 ± 0.18 mg GAE g^−1^ dw while for the control, it was 19.00 ± 0.68 mg GAE g^−1^ dw. Similar increases transpired by applying *t*_pulse_ 10 μs with *T* 1000 μs and *t*_pulse_ 5 μs with *T* 500 μs, which were 29.8% and 29.1%, respectively.

For Exp. Section 3b (Table 2, Exp. Series 13–16), the highest percentage increase was obtained with *t*_pulse_ 20 μs and *T* 1000 μs, namely 32.8% (significant at *p* < 0.05). In particular, the PEF sample resulted in a *Y*_TP_ of 24.57 ± 0.83 mg GAE g^−1^ dw, while for the control, it was 18.50 ± 0.45 mg GAE g^−1^ dw. For the rest of the PEF conditions tested, namely *t*_pulse_ 1 μs with *T* 1000 μs and *t*_pulse_ 5 μs with *T* 1000 μs, lower but significant (*p* < 0.05) increases were observed, particularly 18.4% and 27.2%, respectively.

The outcome from Exp. Sections 3a and 3b indicated the preference for a short pulse period and specifically the set of *t*_pulse_ of 2 μs and *T* of 100 μs. Therefore, these conditions were selected for the next step of the optimization study.

### 3.4. Experimental Section 4 (Exp. Series 17–20)—Optimal Extraction Time 

In Exp. Section 4 (Table 2, Exp. Series 17–20), the effect of the extraction time was quantified as the last parameter of choice for the completion of the PEF assisted OLL extraction optimization. An electric field strength of 0.85 kV cm^−1^, with a *t*_pulse_ of 2 μs and a *T* of 100 μs, was applied for three different extraction times, namely 30, 15, and 10 min. The extraction time of 30 min led to an increase of 35.6% (significant at *p* < 0.05). However, for 15 min treatment duration, an even higher increase (38.9%) was observed (*p* < 0.05). Possibly the exposure of the samples to the air for more time (30 min) increased the oxidation of some compounds. In particular, the *Y*_TP_ for the control sample was 18.30 ± 1.44 mg GAE g^−1^ dw while for the PEF treated extract, *Y*_TP_ reached a 25.35 ± 0.66 mg GAE g^−1^ dw. In contrast to the above trend, the 10 min treatment time proved to be insufficient, having a not significant difference in contrast to the control sample concerning *Y*_TP_. 

From the outcome of Exp. Section 4, the extraction duration of choice for the final verification section of our study was 15 min, half from the initially applied. Such a result has several economical and practical benefits, and it should be utilized at an industrial scale for obvious reasons.

### 3.5. Experimental Section 5 (Exp. Series 21–24)—Verification Section 

Finally, in Exp. Section 5 (Table 2, Exp. Series 21–24), a verification check was performed for the optimal cases found in the previous sections with the difference of applying 15 min instead of 30 min for the extraction duration. As shown in Table 2, the results appear to follow the findings when the treatment time was 30 min. In particular, the highest increase was 34.9% and transpired using *t*_pulse_ of 2 μs and *T* of 100 μs. A similar increment (34.1%) resulted by applying *t*_pulse_ of 1 μs and *T* of 100 μs. The lowest increase was 25.5% when *t*_pulse_ of 10 μs and *T* of 1000 μs were utilized. The percentage increases were not significant between Exp. Series 22 and 23.

### 3.6. Characterization of the Extracts Using HPLC—Polyphenolic Composition of Exp. Series 21–24

**Figure 2:**
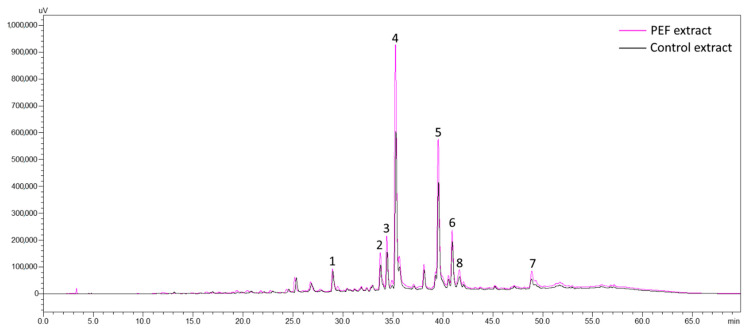
Overlay of chromatograms of PEF and control extracts with *t*_pulse_ 10 μs and *T* 1000 μs. Peak 1: Luteolin diglucoside; Peak 2: Quercetin-3-*O*-rutinoside; Peak 3: Luteolin rutinoside; Peak 4: Luteolin-7-*O*-glucoside; Peak 5: Apigenin-7-*O*-rutinoside; Peak 6: Luteolin-3′-*O*-glucoside; Peak 7: Luteolin aglycone; Peak 8: Oleuropein.

The main components of OLL found during this study are following the literature [6,7,8,9,36,41,42]. In specific, the predominant constituents are oleuropein and luteolin-7-*O*-glucoside. Additionally, luteolin’s related substances are in lower amounts, as well apigenin-7-*O*-rutinoside and quercetin-3-*O*-rutinoside. Seven main compounds were revealed from the chromatogram at 345 nm (Figure 2).

The peaks 2 and 4 with a *λ*_max_ at 349 nm and 345 nm were identified according to their retention time, absorption spectrum, and their corresponding reference substances as quercetin-3-*O*-rutinoside and luteolin-7-*O*-glucoside, respectively. From our previous work [7], peaks 5 and 6 were tentatively identified (by LC-DAD-MS) as apigenin-7-*O*-rutinoside and luteolin-3′-*O*-glucoside, respectively. Due to the lack of corresponding reference substances, peaks 1, 3, and 7 could not be identified, even though according to literature [6,7] and their similar UV-Vis spectrum to luteolin-7-*O*-glucoside, it is believed to be related substances of luteolin. In specific, peak 1 is believed to be luteolin diglucoside which Mylonaki et al. [9] and Herrero et al. [10] identified as luteolin diglucoside eluting barely before quercetin-3-*O*-rutinoside with a *λ*_max_ at 331 nm, just like peak 1 in Figure 2 with a *λ*_max_ at 333 nm. From the findings of Herrero et al. [6], luteolin rutinoside is eluted with a *λ*_max_ at 340 nm between quercetin-3-*O*-rutinoside and luteolin-7-*O*-glucoside. Thus, it is believed that peak 3 is luteolin rutinoside since the chromatogram follows identical elution order with a *λ*_max_ at 345 nm. Peak 8 with a *λ*_max_ at 280 nm was identified as oleuropein based on its retention time and absorption spectrum with the corresponding reference substance. The control sample extracts for the main compounds achieved amounts 0.76 ± 0.03 mg g^−1^ dw for luteolin-7-*O*-glucoside and 0.65 ± 0.04 mg g^−1^ dw for oleuropein (Table 3). Lower amounts were reached by quercetin-3-*O*-rutinoside, apigenin-7-*O*-rutinoside, and luteolin-3′-*O*-glucoside (0.16 ± 0.01, 0.25 ± 0.01, and 0.25 ± 0.03 mg g^−1^ dw, respectively).

**Figure 3:**
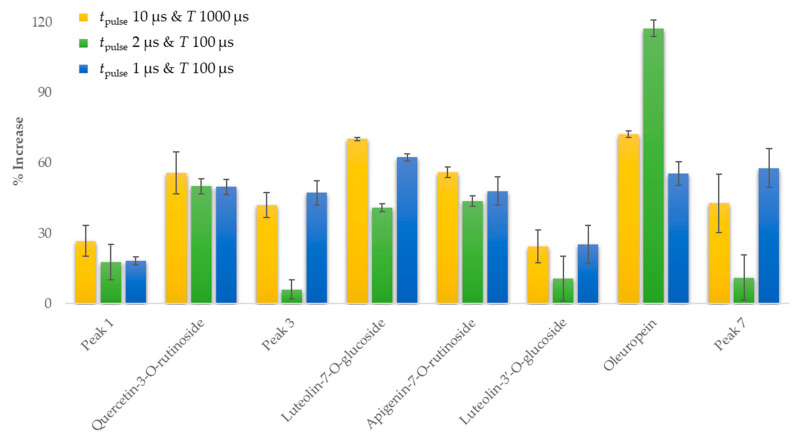
Percentage increases for the main compounds on the three best combinations of *t*_pulse_ and *T*.

It is recognized that the main factors that rule the multitude and the levels of the components detected are the solvent choice, the extraction method, the seasonality, and the locality [43,44,45]. The concentrations of the main identified compounds of OLL extracts between the control sample and three conditions of PEF with 15 min *t*_extraction_ were evaluated to define the PEF treatment effect and especially how different combinations of pulse durations and periods change the polyphenolic composition in the extracts (Table 3, Figure 3).

In specific, the PEF conditions with *t*_pulse_ 10 μs and *T* 1000 μs, *t*_pulse_ 2 μs and *T* 100 μs and *t*_pulse_ 1 μs and *T* 100 μs were examined (for Exp. Series 21–24), while the other PEF parameters were unchanged. In most cases, all the above PEF treatment conditions have shown significant enhancements to the amounts of the tested constituents, which led to an increase up to 117.58%, proving that a disintegration effect on cell membranes of OLL was sufficiently successful even for shorter *t*_pulse_ and *T*. The PEF condition with *t*_pulse_ 10 μs and *T* 1000 μs (Exp. Series 21) reached higher percentage increases than the other two conditions for five of the eight components examined. In specific, for peak 1, quercetin-3-*O*-rutinoside, luteolin-7-*O*-glucoside, apigenin-7-*O*-rutinoside and luteolin-3′-*O*-glucoside was 26.87%, 55.87%, 70.31%, 56.06% and 24.56%, respectively. The rest of the compounds, namely peak 3, oleuropein, and peak 7, ranged from 42.11% to 72.36%. As the PEF treatment with *t*_pulse_ 1 μs and *T* 100 μs (Exp. Series 23) is concerned, it led to a higher increase for peak 3 and peak 7 (47.37% and 57.94%, respectively), while the increment for the other constituents ranged from 18.28% to 62.48%. The highest increase for oleuropein was 117.58% and achieved by the PEF condition with *t*_pulse_ 2 μs and *T* 100 μs (Exp. Series 22). For this condition, the increment for the rest compounds ranged from 6.14% to 50.13%.

For the main compound of OLL, namely luteolin-7-*O*-glucoside, Palmeri et al. [42] achieved an amount of 0.82 mg g^−1^ dw by applying an extraction with water as solvent, high temperature, and a high liquid to solid ratio of 20:1 mL g^−1^. In our work, the same metabolite reached 1.30 ± 0.04 mg g^−1^ dw for the PEF condition with *t*_pulse_ 10 μs and *T* 1000 μs (Exp. Series 21). The significance of this result is based on that a higher amount was gained from a nonthermal effective green extraction method, using a low liquid to solid ratio (3:1). Furthermore, the low percentage (25%) of “green” solvent (EtOH) used, minimizes the cost of its recovery and recycling procedure in the final product and, thus, eliminate the environmental limitations. Additionally, this quantity of luteolin-7-*O*-glucoside appears two times higher than that we have previously reported [36], possibly because of the optimization procedure followed during the work and the different time of collection of OLL (variance in plant material). Concerning the secondary components, quercetin-3-*O*-rutinoside, apigenin-7-*O*-rutinoside, and luteolin-3′-*O*-glucoside, the PEF treatment led to amounts near 0.3 mg g^-1^ dw, where similar concentrations were reached in recent studies [7,42] where high liquid to solid ratios and high energy input to the sample were applied. Finally, for oleuropein, Cifa et al. [46] reached 3.1 mg g^−1^ dw, using a similar percentage of ethanol (30%), somewhat higher liquid to solid ratio (5:1), and ultrasound application for 120 min (energy input range of 12 × 10^3^ kJ kg^−1^). The highest concentration of oleuropein, succeeded with PEF treatment in our work, was 1.41 ± 0.07 mg g^−1^ dw in a much shorter extraction time (15 min) and much lower energy input to the sample (range of 0.1 kJ kg^−1^). However, the results of the above authors are not directly comparable with those of the present work since they have used leaves of a different *O. europaea* L. variety which was also cultivated in different agroclimatic conditions. 

### 3.7. Differential Scanning Calorimetry (DSC)

Antioxidant oxidative stability can be determined using the DSC technique [47]. The estimated temperature at the start of the oxidation process based on observations taken during the incubation period is used to assess this stability. As a result, DSC may be used to deduce oxidation kinetic parameters from the thermographic curves that provide the temperature of the extrapolated initiation of the thermo-oxidation process [48]. *T*_max_, in particular, is the thermographic curve’s highest oxidation peak. The greater the *T*_max_ value, the higher the sample’s resistance.

DSC was used to measure the exothermic peaks of the extracts in this study (Table 4). According to the results, the *T*_max_ was found to be directly related to the total polyphenol content of each sample. The samples of Exp. Series 6 (pulse duration: 10 μs, pulse period: 1000 μs, electric field: 0.85 kV cm^−1^, time of extraction: 30 min) achieved the highest oxidation peak (*T*_max_) of 488 °C (significant at *p* < 0.05), as well as the highest percentage increase (significant at *p* < 0.05) in comparison to the control sample (Exp. Series 8).

## 4. Discussion

The novelty of this study was to deal with optimizing PEF technology as a standalone solid-liquid extraction method for bioactive constituents from freshly cut OLL, under the goal of developing and proposing a method that would replace the conventional extraction techniques by substantially reducing the use of organic solvents and the energy input towards a more efficient, effective, and environmentally friendly polyphenolic compound isolation technique in an economically feasible way.

Overall, PEF enables a higher rate of diffusivity by triggering cell permeabilization changes and, thus, forcing the migration of intracellular components of interest to a solution. Our study design evaluated all the critical parameters affecting the extraction yield of the bioactive compounds comprehensively. The results indicated a significant increase in the total polyphenolic content of the obtained extracts produced using a “green” solvent mixture under fine-tuned PEF conditions.

The amount and nature of the extracted polyphenols depended on the chamber geometry, the applied electric field, the pulse duration and period, and the treatment time; allowing for interesting quantitative and qualitative conclusions over the correlation of the above parameters with the electroporation optimal energy range for the specific plant material cells, the achieved extraction yield and the structure of the extracted metabolites.

The optimal detected PEF contribution on the total polyphenols extractability (38% increase) and constituents of interest for the food, pharma and cosmetic industry (up to 117% increase for specific metabolites) transpired for a rectangular-shaped extraction chamber and 25% *v*/*v* aqueous ethanol solvent choice using a pulse duration (*t*_pulse_) of 2 μs under 0.85 kV cm^−1^ electric field strength (*E*), and a period (*Τ*) of 100 μs for a 15 min extraction duration (*t*_extraction_) ascertaining a significant dependence of PEF assisting extraction performance to the parameters chosen in this study.

Comparing to our previous study [36], for the same raw material (same tree but different season), we reached levels of *Y*_TP_ that resulted from much higher EtOH content solvents (75% EtOH:H_2_O) in half the extraction duration. In particular, during our previous study, we reached 31.45 mg GAE g^−1^ dw with 75% EtOH, *t*_extraction_ of 30 min, and electric field strength of 1 kV cm^−1^, while, in this study, a similar yield (25.49 mg GAE g^−1^ dw) transpired after the optimization of the PEF assisted extraction procedure with only 25% EtOH, 15 min *t*_extraction_, and 0.85 kV cm^−1^ electric field strength. Thus, the achievement is both energy and cost-effective, reducing the cost of the whole process while increasing the environmental friendliness of the process.

From the comparative difference of compound concentration percentage increment on each PEF condition, it appears that PEF conditions (such as *t*_pulse_ and *T*) affect the extraction rate of intracellular components in a nonlinear manner, demonstrating the selectivity of this extraction method. The latter claim is strengthened by the observations and outcome of our previous study [36], where we noticed that *t*_pulse_ affected the extraction rate of identified components, allowing for the selective extraction of distinct OLL molecules. Given that selective extraction is a difficult, time-consuming, and energy-intensive technique, this discovery is critical.

The molecular structure and therefore size, changes in cell membrane breakdown (such as pore size), as well as the solubility of extracted components and the solvent’s polarity, are all possible causes of this selectivity [48,49]. With the exception of oleuropein, the *t*_pulse_ of 10 μs achieved higher or satisfactory increases (no significant difference) for all substances apart from phenolic glycosides, where the molecule usually comprises one or two sugar units bound to a flavone-backbone (quercetin). Because oleuropein’s molecular structure differs from other phenolic compounds in its lack of a flavone backbone and its smaller size, the shorter *t*_pulse_ of 2 μs produced considerably (*p* < 0.05) superior outcomes. Additionally, the different solubility of each component is a crucial factor in PEF treated samples. The solubility of the various components was reported in our previous work [36]. Ιn brief, quercetin-3-*O*-rutinoside has a much lower water solubility than the other compounds, while ethanol is an excellent solvent for oleuropein (all other glycosides are less soluble in it). 

Larger molecules tend to require longer continuous pulse duration for selective extraction. As a result, the key to their optimum selective extraction is a combination of molecular size and solubility.

The results showed that the *T*_max_ was directly related to the total polyphenol content of each sample when using DSC to determine the higher oxidation resistance.

## 5. Conclusions

Based on our findings, the PEF application boosted the performance of conventional static solid–liquid extraction of specific bioactive compounds from fresh olive leaves in an eco-friendly way utilizing green solvents. Even though industrial limitations can originate from the static nature of the standalone extraction optimization proposed technology for continuous flow industrial applications, PEF presents an excellent potential for green selective extraction of polyphenolic compounds from OLL. PEF assisted extraction technology can revitalize functional food manufacturing in a sustainable fashion, generating high-quality products enriched with BACs that have several public health benefits, depending on the biomass qualities, availability, composition, and degree of comminution.

Complementary work is strongly advisable to include the solvent, pH and, polarity effect in the PEF outcome towards maximizing polyphenols concentration. Future work should also focus on further optimization of PEF process parameters to further validate and maximize the selective polyphenols concentration. Another area of future research interest is the influence of chamber content conductivity in the PEF effect.

## Figures and Tables

**Table 1:** PEF process optimization study design.

Exp. Section	Exp. Series	Cell Geometry	*t*_extraxtion_ (min)	*E* (kV cm^−1^)	*t*_pulse_ (μs)	*Τ* (μs)	*N*	*t*_PEFtreatment_ (s)	Energy Input (kWh)	Specific Energy Input (kJ kg^−1^)
1	1	Rectangular	30	1	10	1000	1.80 × 10^6^	18	2.52 × 10^−6^	1.29 × 10^−1^
	2	Rectangular	30	-	-	-	-	-	-	-
	3	Cylindrical	30	1	10	1000	1.80 × 10^6^	18	2.52 × 10^−6^	1.29 × 10^−1^
	4	Cylindrical	30	-	-	-	-	-	-	-
2	5	Rectangular	30	1	10	1000	1.80 × 10^6^	18	2.52 × 10^−6^	1.29 × 10^−1^
	6	Rectangular	30	0.85	10	1000	1.80 × 10^6^	18	2.14 × 10^−6^	1.09 × 10^−1^
	7	Rectangular	30	0.7	10	1000	1.80 × 10^6^	18	1.76 × 10^−6^	9.00 × 10^−2^
	8	Rectangular	30	-	-	-	-	-	-	-
3a	9	Rectangular	30	0.85	10	1000	1.80 × 10^6^	18	2.14 × 10^−6^	1.09 × 10^−1^
	10	Rectangular	30	0.85	5	500	3.60 × 10^6^	18	2.14 × 10^−6^	1.09 × 10^−1^
	11	Rectangular	30	0.85	1	100	1.80 × 10^7^	18	2.14 × 10^−6^	1.09 × 10^−1^
	12	Rectangular	30	-	-	-	-	-	-	-
3b	13	Rectangular	30	0.85	1	1000	1.80 × 10^6^	2	2.14 × 10^−7^	1.09 × 10^−2^
	14	Rectangular	30	0.85	5	1000	1.80 × 10^6^	9	1.07 × 10^−6^	5.46 × 10^−2^
	15	Rectangular	30	0.85	20	1000	1.80 × 10^6^	36	4.28 × 10^−6^	2.19 × 10^−1^
	16	Rectangular	30	-	-	-	-	-	-	-
4	17	Rectangular	30	0.85	2	100	1.80 × 10^7^	36	4.28 × 10^−6^	2.19 × 10^−1^
	18	Rectangular	15	0.85	2	100	9.00 × 10^6^	18	2.14 × 10^−6^	1.09 × 10^−1^
	19	Rectangular	10	0.85	2	100	6.00 × 10^6^	12	1.43 × 10^−6^	7.29 × 10^−2^
	20	Rectangular	30	-	-	-	-	-	-	-
5	21	Rectangular	15	0.85	10	1000	9.00 × 10^5^	9	1.07 × 10^−6^	5.46 × 10^−2^
	22	Rectangular	15	0.85	2	100	9.00 × 10^6^	18	2.14 × 10^−6^	1.09 × 10^−1^
	23	Rectangular	15	0.85	1	100	9.00 × 10^6^	9	1.07 × 10^−6^	5.46 × 10^−2^
	24	Rectangular	15	-	-	-	-	-	-	-

**Table 2:** Mean values of total polyphenol content (mg GAE g^−1^ dw) of OLL extracts.

Exp. Section	Exp. Series	Cell Geometry	*t*_extraxtion_ (min)	*E* (kV cm^−1^)	*t*_pulse_ (μs)	*Τ* (μs)	Average *Y*_TP_ (mg GAE g^−1^ dw) ^1^	SD	% Increase ^1^	SD
1	1	Rectangular	30	1	10	1000	24.98 ^c^	0.56	33.8 ^B^	4.7
	2	Rectangular	30	- ^2^	-	-	18.69 ^b^	1.08	-	-
	3	Cylindrical	30	1	10	1000	16.66 ^a^	1.55	16.0 ^A^	5.5
	4	Cylindrical	30	-	-	-	14.42 ^a^	2.01	-	-
2	5	Rectangular	30	1	10	1000	24.80 ^b^	1.36	29.1 ^A^	2.6
	6	Rectangular	30	0.85	10	1000	26.51 ^b^	0.75	38.1 ^B^	0.8
	7	Rectangular	30	0.7	10	1000	26.30 ^b^	1.28	36.9 ^B^	2
	8	Rectangular	30	-	-	-	19.20 ^a^	0.66	-	-
3a	9	Rectangular	30	0.85	10	1000	24.69 ^b,c^	2.24	29.8 ^A^	2.2
	10	Rectangular	30	0.85	5	500	24.50 ^b^	0	29.1 ^A^	2.6
	11	Rectangular	30	0.85	1	100	24.91 ^c^	0.18	31.2 ^A^	2.8
	12	Rectangular	30	-	-	-	19.00 ^a^	0.68	-	-
3b	13	Rectangular	30	0.85	1	1000	21.90 ^b^	0.42	18.4 ^A^	0.6
	14	Rectangular	30	0.85	5	1000	23.53 ^c^	0.94	27.2 ^B^	2
	15	Rectangular	30	0.85	20	1000	24.57 ^c^	0.83	32.8 ^C^	1.3
	16	Rectangular	30	-	-	-	18.50 ^a^	0.45	-	-
4	17	Rectangular	30	0.85	2	100	24.75 ^b^	0.73	35.6 ^B^	2.7
	18	Rectangular	15	0.85	2	100	25.35 ^b^	0.66	38.9 ^B^	2.4
	19	Rectangular	10	0.85	2	100	17.04 ^a^	0.21	−6.6 ^A^	2.2
	20	Rectangular	30	-	-	-	18.30 ^a^	1.44	-	-
5	21	Rectangular	15	0.85	10	1000	23.71 ^b^	0.29	25.5 ^A^	3.1
	22	Rectangular	15	0.85	2	100	25.49 ^c^	0.88	34.9 ^B^	0.3
	23	Rectangular	15	0.85	1	100	25.34 ^c^	1.1	34.1 ^B^	0.9
	24	Rectangular	15	-	-	-	18.90 ^a^	0.69	-	-

^1^ Means within rows of each Exp. Section with different superscript letters (a–c; A–C) are significantly (*p* < 0.05) different. ^2^ “-“ denotes no values for control samples (no PEF applied).

**Table 3:** Major compounds concentration (mg g^−1^ dw) of OLL extracts prepared with 25% aqueous ethanol for extraction duration of 15 min.

Exp. Series	Concentration Parameters	Peak 1 ^1^	Quercetin- 3-*O*- Rutinoside	Peak 3 ^1^	Luteolin- 7-*O*- Glucoside	Apigenin- 7-*O*- Rutinoside ^2^	Luteolin- 3′-*O*- Glucoside ^1^	Oleuropein	Peak 7 ^1^
21	Average ^3^	0.14 ^a^	0.25 ^b^	0.27 ^b^	1.30 ^c^	0.39 ^c^	0.31 ^b^	1.12 ^c^	0.09 ^b^
	SD	0.02	0.03	0.01	0.04	0.01	0.02	0.06	0
	% Increase ^3^	26.87 ^B^	55.87 ^A^	42.11 ^B^	70.31 ^C^	56.06 ^B^	24.56 ^A^	72.36 ^B^	42.86 ^B^
	SD	6.68	9.03	5.26	0.72	2.24	7	1.38	12.37
22	Average	0.13 ^a^	0.24 ^b^	0.20 ^a^	1.08 ^b^	0.36 ^b,c^	0.28 ^a^	1.41 ^d^	0.07 ^a^
	SD	0.02	0.01	0.01	0.05	0.02	0.01	0.07	0
	% Increase	17.73 ^A,B^	50.13 ^A^	6.14 ^A^	41.02 ^A^	43.94 ^A^	10.73 ^A^	117.58 ^C^	11.11 ^A^
	SD	7.51	3.14	4.02	1.6	2.24	9.52	3.46	9.62
23	Average	0.13 ^a^	0.24 ^b^	0.28 ^b^	1.24 ^c^	0.37 ^b^	0.31 ^b^	1.01 ^b^	0.10 ^b^
	SD	0.01	0.02	0.01	0.03	0	0.02	0.03	0.01
	% Increase	18.28 ^A^	49.87 ^A^	47.37 ^B^	62.48 ^B^	48.16 ^A,B^	25.32 ^A^	55.59 ^A^	57.94 ^B^
	SD	1.67	3.14	5.26	1.5	5.93	8.2	4.97	8.36
24	Average	0.11 ^a^	0.16 ^a^	0.19 ^a^	0.76 ^a^	0.25 ^a^	0.25 ^a^	0.65 ^a^	0.06 ^a^
	SD	0.01	0.01	0	0.03	0.01	0.03	0.04	0.01
	% Increase	- ^4^	-	-	-	-	-	-	-

^1^ Luteolin-3′-*O*-glucoside as well as peaks 1, 3 and 7 were quantified as luteolin-7-*O*-glucoside. ^2^ Apigenin-7-*O*-rutinoside was quantified as apigenin. ^3^ Means within each column (compound) with different superscript letters (a–c; A–C) are significantly (*p* < 0.05) different. ^4^ “-“ denotes no values for control samples (no PEF applied).

**Table 4:** DSC results on the oxidation temperature (*T*_max_) of the various samples.

Exp. Section	Exp. Series	PEF Treated Extract		Exp. Series	Control Extract		% Increase ^1^	SD
		Average Oxidation Temperature (°C) ^1^	SD		Average Oxidation Temperature (°C) ^1^	SD		
1	1	476 ^a^	1	2	415 ^b^	1	14.78 ^B^	0.81
	3	411 ^c^	2	4	401 ^c^	1	2.49 ^A^	0.24
2	5	475 ^a^	1	8	418 ^d^	2	13.63 ^A^	0.89
	6	488 ^b^	2				16.82 ^B^	0.76
	7	484 ^c^	1				15.95 ^B^	0.53
3a	9	473 ^a^	1	12	416 ^c^	1	13.70 ^A^	0.67
	10	472 ^a^	1				13.46 ^A^	0.69
	11	477 ^b^	1				14.66 ^A^	0.56
3b	13	459 ^a^	2	16	414 ^d^	2	10.95 ^A^	0.19
	14	468 ^b^	1				13.04 ^B^	0.18
	15	474 ^c^	1				14.57 ^C^	0.09
4	17	475 ^a^	2	20	414 ^d^	2	14.73 ^B^	0.65
	18	480 ^b^	1				15.94 ^B^	0.89
	19	412 ^c^	1				−0.48 ^A^	0.24
5	21	469 ^a^	2	24	416 ^c^	1	12.74 ^A^	0.21
	22	482 ^b^	1				15.87 ^B^	0.24
	23	480 ^b^	2				15.38 ^B^	0.2

^1^ Means within rows (Exp. Sections) with different superscript letters (a–d; A–C) are significantly (*p* < 0.05) different.

## Data Availability

The data presented in this study are available in this manuscript.
